# Loss of Genetic Redundancy in Reductive Genome Evolution

**DOI:** 10.1371/journal.pcbi.1001082

**Published:** 2011-02-17

**Authors:** André G. Mendonça, Renato J. Alves, José B. Pereira-Leal

**Affiliations:** Instituto Gulbenkian de Ciência, Oeiras, Portugal; Weizmann Institute of Science, Israel

## Abstract

Biological systems evolved to be functionally robust in uncertain environments, but also highly adaptable. Such robustness is partly achieved by genetic redundancy, where the failure of a specific component through mutation or environmental challenge can be compensated by duplicate components capable of performing, to a limited extent, the same function. Highly variable environments require very robust systems. Conversely, predictable environments should not place a high selective value on robustness. Here we test this hypothesis by investigating the evolutionary dynamics of genetic redundancy in extremely reduced genomes, found mostly in intracellular parasites and endosymbionts. By combining data analysis with simulations of genome evolution we show that in the extensive gene loss suffered by reduced genomes there is a selective drive to keep the diversity of protein families while sacrificing paralogy. We show that this is not a by-product of the known drivers of genome reduction and that there is very limited convergence to a common core of families, indicating that the repertoire of protein families in reduced genomes is the result of historical contingency and niche-specific adaptations. We propose that our observations reflect a loss of genetic redundancy due to a decreased selection for robustness in a predictable environment.

## Author Summary

Bacteria have found many niches in which to live, and one of them is inside eukaryotic cells. These intracellular bacteria include endosymbionts like *Buchnera aphidicola*, which provides its host, an aphid, with essential amino acids, as well as many pathogenic bacteria such as *Mycobacterium leprae* and *Rickettsia prowazekii*, the causative agents of leprosy and typhus, respectively. Even though they all evolved their intracellular lifestyle independently, all these bacteria lost a large number of genes as they adapted to their hosts, presumably because the rich environment where they found themselves no longer required such functions. For example, biosynthetic genes are frequently lost. It has been a matter of debate what decides whether a gene can be lost in evolution, and intracellular bacteria have been used as model systems to study these processes. In our study, we propose that when adopting an intracellular lifestyle, these bacteria extensively lost duplicated genes. We propose that this represents loss of copy redundancy that is possible because the host cell represents a predictable environment in which there is little pressure for the bacteria to retain these backups. In simplistic terms, if the road is always smooth, you are probably OK without a spare tire.

## Introduction

Living organisms evolved to be functional in frequently harsh and variable environments, buffering internal molecular noise, genetic variation and unpredictable environmental fluctuations. Such ability is termed robustness [1]. One common source of robustness is genetic redundancy, in which one or more genes can perform the same function [2]. The exact contribution of genetic redundancy to the robustness of biological systems has, however, been a subject of considerable debate. On the one hand, it is hard to understand how full redundancy can be evolutionarily stable. After duplication the two copies will have identical functions and the loss of one by the accumulation of mutations is buffered by the other, having no fitness cost [3].

On the other hand, there is strong evidence for functional redundancy by duplicates. The deletion of singleton genes, *i.e.*, those without copies, is frequently lethal [4]. In contrast, deletion of genes with paralogues has frequently little fitness cost [4], even though the deletion of pairs of paralogues has frequently high fitness costs [5], suggesting that their compound function is essential, and arguing for functional redundancy of the paralogues. The capacity for functional compensation correlates with sequence divergence, with closer paralogues more likely to provide it [4], which argues for gene duplication providing functional redundancy. This redundancy can in fact be maintained over large periods of time, as two independent studies of functionally redundant duplicates showed recently [6], [7]. A theoretical analysis of the metabolic network of *S. cerevisiae* estimated that the dispensability of up to 28% of metabolic enzymes can be attributed to the existence of a compensating paralogue [8]. Recent work has suggested that the cost of maintenance of complete redundancy can be, to some extent, offset by partial functional redundancy [9]. Furthermore, incomplete and presumed functionally distinct duplicates may also provide additional backup [10].

The discussion on the role of genetic redundancy in robustness is also centered on the conditions for the emergence of robustness. A series of theoretical studies have resulted in the prediction that high robustness can only evolve in the presence of frequent perturbations (reviewed in [11]). Little attention has been given to the conditions necessary for the loss of robustness. Based on the above, we would anticipate that predictable environments should not place a high selective value on robustness. The intracellular environment is relatively invariant over time. Organisms that occupy this ecological niche are not subjected to repeated nor frequent perturbations, and represent a good system to study adaptation to such predictable environments. The rapid increase in the number of sequenced intracellular endosymbionts and parasites provides an ideal system to study the evolution of genetic redundancy, and for an empirical study on the importance of external perturbation in the emergence of robustness.

Intracellular lifestyles have been frequently and independently adopted by bacteria and eukaryotes, in the context of endosymbiosis or parasitic relationships [12]–[16]. Obligate intracellular parasites and endosymbionts have committed to an intracellular lifestyle, only capable to replicate inside a host eukaryotic cell [14]. They include organisms like *Buchnera aphidicola*, a bacterial endosymbiont of aphids and also parasitic, pathogenic bacteria, such as *Mycobacterium leprae* and *Rickettsia prowazekii*, the causative agents of leprosy and typhus, respectively. The adaptation to the intracellular niche is invariably accompanied by extensive gene loss [12]–[14]. Reduction in gene repertoires is believed to be associated with adaptation to a new lifestyle where many molecules can be obtained from the host [17]. Since the host provides metabolites, many loci in the endosymbiont/parasite would become redundant and previously deleterious mutations would become de facto neutral, due to relaxed selection. Examples are the loss of biosynthetic pathways in many endosymbionts (e.g. [15]). A second driver of gene loss is the drastic reduction in effective population sizes [18]–[20], associated with high mutation rates [21]. Furthermore, inheritance modes of intracellular bacteria imply that only few individuals are transmitted across generations and/or hosts, generating repeated population bottlenecks [15]. Even “important” genes involved in DNA repair, transcriptional regulation and replication have been lost in *Buchnera*, suggesting that drift plays an important role in genome reduction [22]–[25]. Extreme reductive genome evolution is also observed in obligate parasitic bacteria like the Mycoplasmas, which are often described as the simplest self-replicating organisms [26]. These organisms are obligate parasites of vertebrates, living under an invariant environment within the hosts. We consider these organisms, together with obligate intracellular parasites and endosymbionts, as “Reduced genomes” living under predictable environments.

Here we study the dynamics of gene loss in Reduced genomes, investigating which genes can be lost, and find a previously undescribed driver for gene loss. By combining data analyses with evolutionary simulations we find empirical evidence for a selective drive to maintain diversity of protein families at the expense of family size, with the emergence of many genes without any paralogues. We propose that the latter represents a loss of genetic redundancy due to a decreased selection for robustness in a predictable environment.

## Results

### Protein family dynamics in Reduced genomes

Protein families represent groups of proteins that share a common evolutionary history [27]. Within protein families there is conservation of structure and biochemical function across large evolutionary distances [28]. The number of protein families can be construed as the degree of information coded in a genome – the more distinct families exist, the more information. Early analysis of a small number of completely sequenced genomes suggested that larger genomes have more protein families than smaller ones [29]–[31], and there is, in fact, a linear relationship between the number of genes and number of protein families [29]. Larger genomes also tend to have larger protein families [30], [31]. Furthermore, intracellular parasites and endosymbionts that have the smallest genomes known, also have the smallest gene families [31]. With the accumulation of completely sequenced genomes of bacterial parasites and endosymbionts we can now address whether these reduced genomes living under nearly constant environments display the same use of protein families. We chose to define protein families based on structural domain architectures [32], which provides a higher sensitivity than other sequence-based methods [33] and allows us to capture distant evolutionary relationships. Members of each family should be traceable to a common ancestor by duplication and speciation [27], [34]. Note that in bacteria, Lateral Gene Transfer is frequent and generates copies of genes (xenologues) that are indistinguishable from copies resulting from duplication (paralogues) [35]–[37]. For the purpose of this analysis, their specific origin is not relevant and we use the term paralogue loosely to include both.

**Figure 1:**
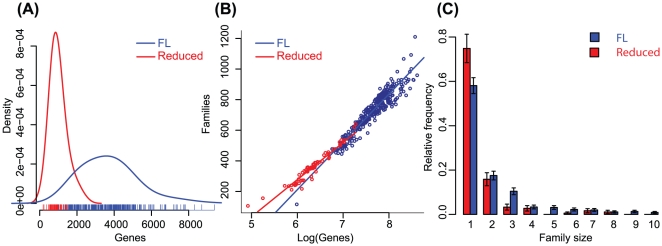
Reduced paralogy in reduced genomes. (**A**) Genome size distribution in FL (Blue) and Reduced (Red) bacteria, measured as number of genes. We computed a density function (solid lines), and the short colored lines on the x-axis represent the individual genomes. The data set of 69 Reduced genomes has significantly fewer genes (990±439) than the free-living set of 308 genomes (3695±1479: p<2.2.×10^−16^, Mann-Whitney U test). (**B**) Reduced genomes have a higher number of families per gene than FL. Solid lines represent a fit by a logarithmic function (r^2^ = 0.95). The slopes are statistically different (p<4.3×10^−7^) (**C**) Reduced genomes have a higher number of singletons and smaller number of protein families with more than one element. These distributions are significantly different (p = 0.00018, χ^2^ homogeneity test on absolute frequencies). Note that because of the variability of the size of the families in different genomes, we subjected it to a high pass filter (see methods).

We studied 69 bacteria that have undergone extensive reductive genome evolution that we label “Reduced”, consisting of the obligate parasitic mycoplasmas and obligate intracellular parasites and endosymbionts, and 308 Free living bacteria, which we label “FL”. In our analysis these two classes are mutually exclusive and their genome size distribution significantly different (**Figure 1A**). Species name are provided in **tables S3 and S4 in Text S1**. As expected we observed a strong positive correlation between the number of genes and families (Spearman's rank correlation ρ = 0.97). We noted however that there were two statistically distinct trends in FL and Reduced organisms (**Figure 1B**). Reduced genomes have more families than would be expected if they were part of the FL. The same trend is observed when we consider individual protein domains instead of protein families (**Figure S1 in Text S1**). Because the number of genes and families in the two populations are very different and hence difficult to compare, we tested the potential difference between the two populations of organisms by estimating the elasticity of each population, a measure that captures the responsiveness of a function to parameters in a relative scale. The elasticity of Families in Reduced genomes is two times higher when compared to FL. In other words, adding one gene is 50% more likely to drive a number of families increase in Reduced than in FL. Technically, a 1% change in the number of genes will determine a variation of 0.73% in the number of families, compared to a variation of 0.48% in FL genomes. Thus FL genomes are more robust to gene number variation than are Reduced genomes.

**Figure 2:**
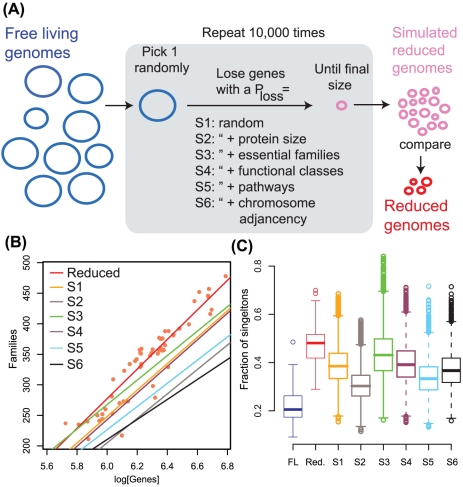
Selective loss of paralogy. (**A**) Cartoon illustrating the simulation of gene loss according to different scenarios. (**B**) Protein family diversity is higher in Reduced genomes than in the simulated reduced genomes. Individual genomes (open circles) and the logarithmic fit for the Reduced genomes is shown in red. Only the logarithmic fit is shown for each simulation (r^2^_S1_ = 0.91; r^2^_S2_ = 0.84; r^2^_S3_ = 0.93; r^2^_S4_ = 0.91, r^2^_S5_ = 0.86; r^2^_S6_ = 0.89). The slopes of the fits are significantly lower than those of the Reduced genomes (*p*_S1_ = 0.006, *p*_S2_ = 2.1×10^−4^, *p*_S3_ = 1.6×10^−7^, *p*_S4_ = 0.003, *p*_S5_ = 2.82×10^−17^, *p*_S6_ = 3.08×10^−9^ t-test) (**C**) The plot shows the relative frequency of singletons in FL, Reduced and simulated genomes. Reduced genomes have more singletons than could be expected under any of the simulated scenarios (p_S1_ = 0.002, p_S2_ = 0.0004, p_S3_ = 0.005, p_S4_ = 0.002, p_S5_ = 0.0008, p_S6_ = 0.002, Mann-Whitney U test).

Smaller genomes, such as those found in intracellular parasites and endosymbionts, were previously shown to have smaller families [30], [31]. Our results reveal that Reduced genomes had smaller families than could be expected if they followed the same trend as the FL genomes, in particular, they had a significantly higher number of singletons, *i.e.* families of size one (**Figure 1C** - note that family size has been subjected to a high pass filter - see methods for details). These results hold when these comparisons are made only for organisms within the same order, which suggests that phylogenetic distance is not an important bias in this result (**Figures S2, S3, S4 and S5 in Text S1**). A simple averaging of the fraction of singletons in both populations illustrates this trend well - 22% of the families in FL are singletons, but this number rises to 48% in Reduced genomes (p<2.2×10^−16^; Mann-Whitney U test, **Figure 2C**). Another way to look at the same problem is to compute the number of genes in paralogous families [31] (**Figure S6 in Text S1**). As before, phylogenetic distance does not bias this result (**Figure S7 in Text S1**). Note that although gene loss is the dominant force accounting for the difference in size between Reduced and Free Living families, there is also gene duplication in Free living organisms, and what we measure is the compound signal of both FL duplication and Reduced loss.

Taken together these observations indicate that the reduced genomes are not a random sample of the FL genomes. They have relatively more distinct protein families than FL genomes but less elements per family, which suggests a selective drive to keep diversity of protein families at the expense of redundancy. This is the hypothesis we will test here.

### Neutral models of gene loss

In order to claim that protein diversity or redundancy are selectively lost and/or retained we first need to determine whether this is not the outcome of a neutral process, or that it is not the byproduct of a selective drive on some other character. To address these points we modeled gene loss under a variety of scenarios. We considered two scenarios modeling neutral gene loss and two capturing functional selection. The details of the simulation are described in the methods section, and summarized in **Figure 2A**. In short, we randomly sample the FL genomes and then simulate gene loss up to a final genome size according to predefined scenarios, where the key variable between scenarios is the probability of losing each gene. We run the simulations 10,000 times for each scenario, creating populations of simulated reduced genomes that we then compare with the Reduced set.

We first simulated two independent scenarios of neutral gene loss. In the first scenario (S1) genes to be lost are randomly sampled and have a constant probability of loss that corresponds to the average difference in number of genes in the genome between FL and Reduced genomes. A second, more sophisticated scenario accounts for the fact that longer genes may receive more mutations, which we simulate in scenario S2 by tying the probability of gene loss to its size. Neutral loss would result in significantly lower protein family diversity than observed in the Reduced genomes (**Figure 2B**). For example, a Reduced genome with 1000 genes would have 510 families, whereas simulated genomes under scenarios S1 and S2 would have 449 and 398 families respectively. Moreover, neutral loss would result in significantly fewer singletons than we observe in Reduced genomes, i.e. higher genetic redundancy (<39%, compared to 48% in Reduced - **Figure 2C**). These results hold even when we we perform the simulations within the same bacterial order, which indicates that our results are robust to the large phylogenetic distances considered (**Figure S8 in Text S1**).

From this we conclude that neutral gene loss alone cannot account for the observed diversity of protein families, nor for the reduced genetic redundancy. Rejection of a neutral scenario is suggestive of selection but does not allow us to determine what is being selected. In other words, we cannot state that there is selection for protein family diversity or against redundancy as it is altogether plausible that there is selection on some completely unrelated character and what we observe is the byproduct of that selective drive. The genes preferentially conserved could be enriched in specific protein families, thus biasing our results. We now consider the major factors that can constrain gene conservation, and by extension its loss.

### Models of selective gene loss

We now investigate the possibility that there is preferential retention of a subset of genes on some functional grounds that incidentally result in retention of protein diversity. We first consider that Essential genes may define such set of genes that are preferentially retained, where essential genes are defined by having a lethal gene deletion phenotype. Essential genes in bacteria are preferentially retained in evolution [38], [39]. In eukaryotes essential genes have a lower probability of being lost in the context of lineage-specific gene loss [40]. We observed, as expected, that essential genes in *E. coli* are preferentially conserved in bacterial parasites and endosymbionts (**Figure S9 in Text S1**). Note that these genes can still be lost, as is well illustrated by experimental evolution studies of genome reduction in *Salmonella enterica* where essential genes were in fact lost [41]. In scenario S3 we thus preferentially keep protein families that have essential genes in *E. coli*, *i.e.* we consider essentiality a property of the family [42].

Although genes are lost in all categories, some functional classes are preferentially lost and others preferentially retained [13], [15], [43]. We calculated the functional class distributions in both populations, and observed several statistical significant differences, for example a preferential retention of genes annotated to the functional class Translation (**Figure S10, Table S1 and S2 in Text S1**). In scenario S4 we preferentially retain protein families annotated to the most abundant functional classes in the Reduced genomes.

Proteins do not work in isolation but they establish interactions and form pathways, and this could constraint the probability of gene loss. We consider participation in metabolic pathways as these can be inferred from sequence alone with reasonable confidence, and physiological coupling in pathways was shown to be a constraint in reductive genome evolution, *i.e.* coupled reactions are more likely to be lost together [44]. We simulate gene loss in a scenario where once a member of a pathway is lost, the probability of losing other members of the pathway increase three-fold (**S5**). Protein-protein interactions may also play a role in gene retention, however we lack the data to address these interactions, and it is unclear at which evolutionary distances it is safe to transfer protein-protein interactions. Furthermore, there is conflicting evidence regarding the role they can play in gene loss. Ochmann and co-workers found that poorly connected proteins are more likely to be lost in the evolution of γ-proteobacteria [45], while Tamanes and co-workers found that in the reductive evolution of *Buchnera aphidicola APS*, gene loss did not correlate with the absolute number of links of a protein in the protein interaction network (some hubs were more likely to be preserved than others), nor did they observe any drive to keep functional modules intact [46].

We consider a final scenario where gene positioning can determine the likelihood of a gene being lost, as larger deletions could simultaneously delete more than one gene. This has been in fact proposed to be frequent for example in the evolution of *B. aphidicola* [47] and of *Burkholderia mallei* [48], even though other studies suggests that loss of individual genes may also be frequent [49]. Note that the organization of bacterial chromosomes in operons makes this scenario also pertinent to understand functional constraints to gene loss, as genes that are part of the same operon likely code to proteins that are functionally associated, as part of the same pathway, complex or directly interacting with each other, and gene order is frequently conserved [50]. We thus modeled a final scenario (**S6**) where once a gene is lost, adjacent genes become twice as likely to be lost.

Comparison of these selective loss scenarios with the Reduced genomes indicates that selection based on predicted essentiality, functional classes, co-participation on predicted metabolic pathways or adjacency in the genome cannot account for the increased protein family diversity observed, which is substantially higher than observed in the simulations. Using the same example as above, simulated genomes with 1000 genes would have S3 = 464, S4 = 453, S5 = 386 and S6 = 431 families, compared to the 510 families in Reduced genomes. Furthermore, none of these simulations can produce singleton numbers as high as observed in reduced genomes (S3 = 43%, S4 = 39%, S5 = 34%, S6 = 37% compared to 48% in Reduced - **Figure 2C**. Thus, although all the factors we tested can constraint gene loss, our simulations indicate that they cannot account for the protein family diversity nor the reduction in genetic redundancy we observe in Reduced genomes.

### Convergence to a common core?

Genome reduction happened multiple independent times in the course of evolution, but it is plausible that there is convergence to a particular small set of genes necessary for parasitic or endosymbiotic life. Such convergence to a minimal gene set could represent a constraint to gene loss accounting for some of the protein family diversity we observe in the Reduced genomes. Previous attempts to define minimal gene sets compatible with cellular life using orthology, revealed a small number of genes [26], [51]. This lead to the proposal that non-orthologous gene displacement, where the same function is performed by unrelated or very distantly related, non-orthologous proteins [52], was far more important than previously anticipated [26]. In fact a comparison of the shared homologous protein coding genes between endosymbionts and the parasite *Mycoplasma genitalium* revealed a small set of 175 homologous groups that could represent the minimal core for cellular life [24].

**Figure 3:**
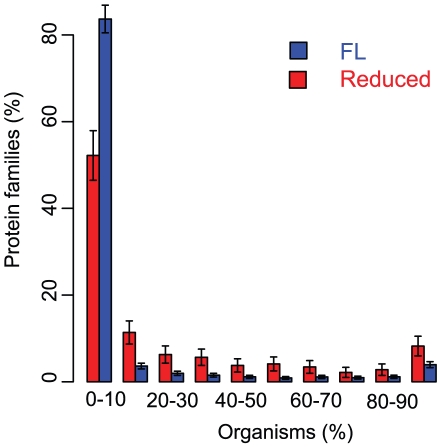
Conservation of protein families by fraction of the organisms. Only a restricted core of families is present in the majority of organisms in both Reduced and FL. The two distributions are significantly different at *p*<2.2×10^−16^ (χ^2^ homogeneity test).

Using a sensitive protein family detection method we now ask whether we can detect the convergence to a common set of protein families in the genomes we analyzed here. This could represent a minimal core of families necessary for parasitic and/or endosymbiotic life. We found that only a small proportion (8%) of the families observed in Reduced genomes is present in more than 90% of the organisms (118 protein families in 1433). Similarly, only 4% of the FL families are present in more than 90% of these organisms (293 out of 7405 - **Figure 3**). The 118 families common to Reduced organisms are a subset of the families common to FL organisms. This suggests that the common core of families necessary for parasitic or endosymbiotic life is a subset of those necessary for free life. Note that only 43% of the protein families retained in most Reduced genome are essential in *E. coli* (51/118), which further strengthens the idea that each ecological niche requires distinct sets of proteins families. Note that this small number is not due to the existence of two distinct life styles in the Reduced group, as when we break this group into parasites and endosymbionts, we observe a only marginal increase in the number of families that are present in more than 90% of the organisms (132 in parasites and 162 families in endosymbionts). In contrast, we find that most families are present in less than 10% of the organisms. In both populations the majority of the protein families falls into this group, but these “unique” families are more common in FL (84%) than in Reduced genomes (52%). From this we can extrapolate that although niche- and taxon-specific adaptations dominate Reduced genomes, they are comparably less important than in FL organisms.

Thus, convergence to a common core of protein families does not appear to be a major force shaping the protein family diversity in reductive genome evolution.

### Loss of genetic redundancy

Our results so far are compatible with a scenario where there is a selective drive to retain a minimal set of families compatible with life in the specific niche occupied by the organism, and that this includes a small core of families common to all reduced genomes, as well as retention of specific functions. This results in the measured increase in protein family diversity in Reduced genomes. However, none of the neutral and selective scenarios we modeled or analyzed above can account for the marked reduction in protein family size, in particular the increase in the number of singletons in Reduced genomes. We hypothesize that this observation may be explained by loss of genetic redundancy, i.e. when more than one gene can perform the same function in a free living organism (larger families), those copies will be lost in the course of reductive genome evolution up to a point where only a single gene per function is retained (singletons). There are abundant anecdotal evidence that supports this hypothesis. For example, most Bacteria have two peptide chain release factor proteins with partial overlap in codon specificity (PrfA: UAG,UAA; PrfB: UGA,UAA). *Legionella Pneumophila*, a pathogenic γ-proteobacteria that is a facultative parasite, has even a third member of this family (lpg0167); in contrast the related intracellular parasite *Coxiella burnetii*, the causative agent of Q fever, retained only PrfB.

**Figure 4:**
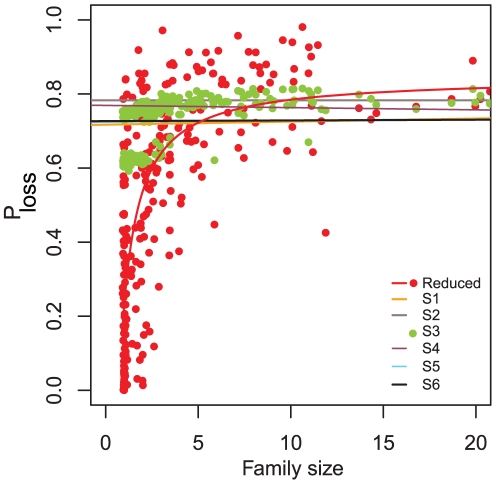
Gene loss depends on degree of paralogy. Probability of gene loss in families present in most reduced genomes (Red points) shown as a function of protein family size. The Red line is an inverse function fitted to the Reduced families. Colored lines represent the results of the different simulations, as indicated in the lower right corner, showing that under these scenarios P_loss_ is independent of family size. The Green points are the Reduced families simulated under scenario S3, showing that although there are two distinct P_loss_ levels hardwired into the simulation, they are both independent of family size.

This scenario requires then that larger families are more likely to lose genes than smaller ones. We tested this hypothesis and found that the probability of gene loss in families present in most organisms is positively correlated with family size (Spearman's rank correlation ρ = 0.74) (**Figure 4**). This relationship is best approximated by an inverse function (r^2^ = 0.55), which suggests that the probability of gene loss is essentially random for larger families, but as families become smaller it decreases sharply, with small families having very small probabilities of gene loss. The probability of a gene being lost thus depends on the number of paralogues it has. In neutral scenarios such positive correlation is absent (ρ_S1_ = 0.04, ρ_S2_ = −0.32), and is also absent in the scenario where we retain specific functional classes (ρ_S4_ = 0.07), members of the same pathway (ρ_S5_ = 0.06) or adjacent genes (ρ_S6_ = 0.06). In scenario S3 we observed a correlation between family size and probability of gene loss (ρ_S3_ = 0.74), but inspection of the data in **Figure 4** suggests that this is an artifact resulting from hardwiring two distinct levels of Probability of loss in the simulation.

Are the genes being lost those that were functionally redundant with their paralogues? Anecdotal evidence suggest that this is the case. There are for example at least seven Cof-like phosphatases in *E.coli* (Cof, YidA, YbhA, YigL, YbiV, YbjI, YedP), with substantial overlap in their substrate specificities . In contrast, the endosymbiont Candidatus *Blochmannia pennsylvanicus* has a single gene assignable to this family (YigL), which is predicted to maintain 4 out of the 5 substrates that the different *E.coli* enzymes are known to process [53], [54]. Is this a general case? To answer this question we struggle with the absence of extensive functional information for most of the organisms studied here, the varying phylogenetic distances between these organisms and difficulty of large-scale mapping of orthologues in paralogous families.

**Figure 5:**
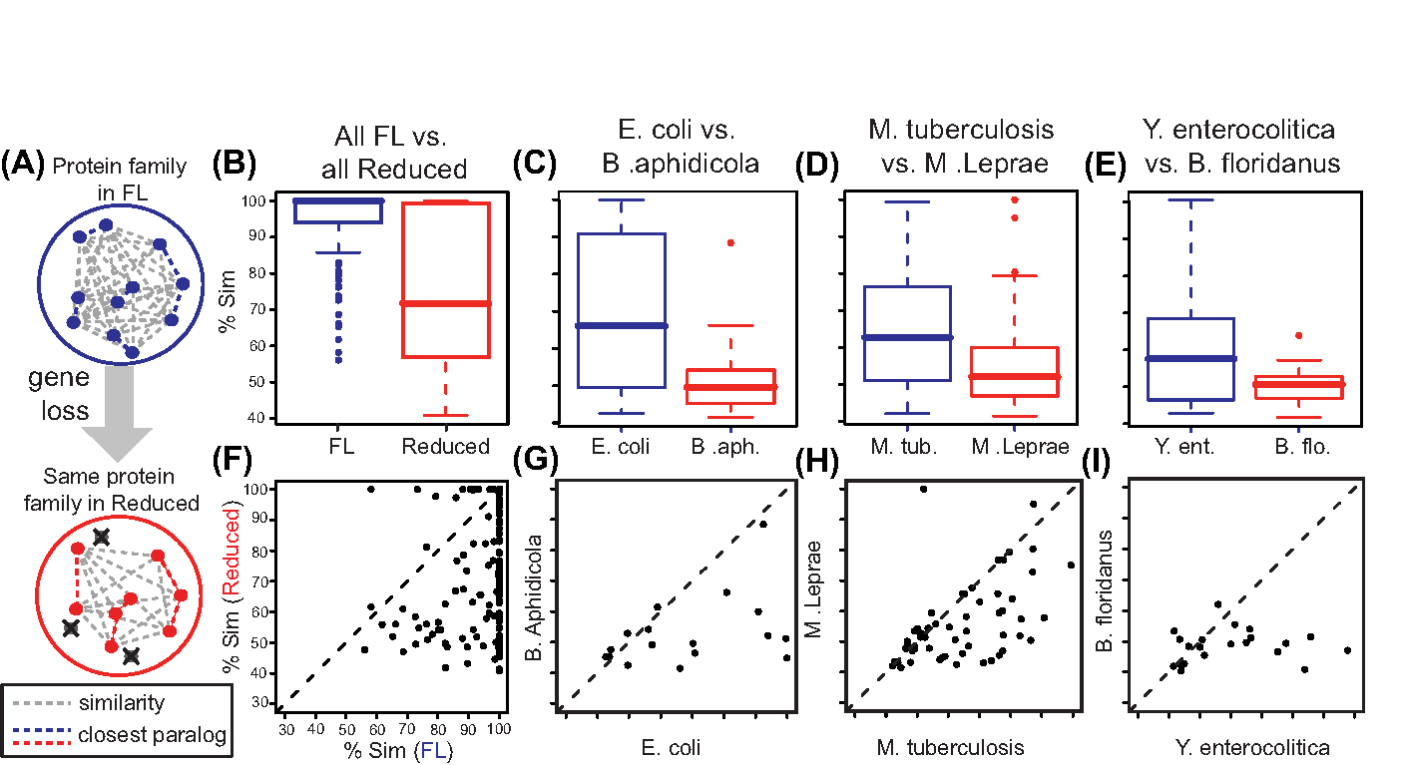
Closest paralogues are preferentially lost. (**A**) Cartoon representing the similarity network in one protein family in free living organisms blue circle, with the most similar paralogues highlighted (in blue lines). Selective gene loss in reductive genome evolution removes preferentially those genes that have the most similar paralogues resulting in a Reduced family similarity network (in red), where the closest pairs of paralogues are more dissimilar. Thus the prediction is that the closest pairs of paralogues in FL are more similar than the closest pairs in Reduced. The similarity between the closest paralogues in the similarity network in each protein family is significantly lower in Reduced genomes, when (**B**) all genomes are considered (p = 1.1×10^−22^, Wilcoxon test), as well as for phylogenetically related organisms (**C–E**, p = 0.03, p = 0.002, p = 0.04, respectively). (**F**) In 89.7% of the protein families, the closest pair of paralogues is more similar in FL than in Reduced genomes, and the same is true for the same example organisms considered (**G–I**, 71%, 71% and 76%, respectively). Species represented are FL: *Escherichia coli*, *Mycobacterium tuberculosis*, *Yersinia enterocolitica*, Reduced*: Buchnera aphidicola*, *Mycobacterium leprae*, Candidatus *Blochmannia floridanus*.

We first seek to address the issue of functional redundancy in a way that does not require such mappings nor functional information, by focusing on the most similar pairs of paralogues [4], [10]. The rational of our experiment is the following: if the most similar pairs of paralogues in a protein family are the ones that are more likely redundant, then one member of the pair will be preferentially lost in Reduced genomes, resulting in a decrease in the similarity between the pairs of paralogues in the family. Thus, we computed the sequence similarity between the pairs of closest paralogues for each family and within each genome (**Figure 5A**). We observed that the pairs of closest paralogues in the families in Reduced genomes are significantly less similar than those in the FL genomes (**Figure 5B**). We detected a reduction in the similarity of the closest paralogues in nearly 90% of the protein families (**Figure 5F**). Note that this is not an artifact of the increased sequence divergence in Reduced genomes, as we control for this - in fact, the overall sequence similarity within Reduced families is higher than in the same FL families (not shown). Furthermore, those families that did not reduce in size do not display this reduction in similarity (**Figure S11 in Text S1**). Additionally, the difference in family size could bias this analysis, but when we control for it we show that the reduction in similarity still holds (**Figure S12 in Text S1**). This analysis is also potentially biased by phylogenetic distance between organisms compared and different sizes of the universes being compared. However, when we consider specific pairs of phylogenetically close FL and Reduced organisms, i.e. one-to-one comparisons, we find the same trend (**Figures 5C, D, E, G, H, I**). Thus, reductive genome evolution results in the increasing of the distance between the closest paralogues, which we interpret as evidence that there is preferential loss of one of the pair of closest paralogues.

One example of this scenario is the protein family that includes in *E. coli* the two redundant transketolases TktA and TktB (E.C. 2.2.1.1) [55], [56], as well as the functionally distinct Dsx (1-deoxyxylulose-5-phosphate synthase, E.C. 2.2.1.7). TktA and TktB are 99% identical, but only 29% identical to Dsx. In the closely related *B. aphidicola*, only one transketolase was retained (Tkt), together with the Dsx ortholog - they are ∼13% identical.

**Figure 6:**
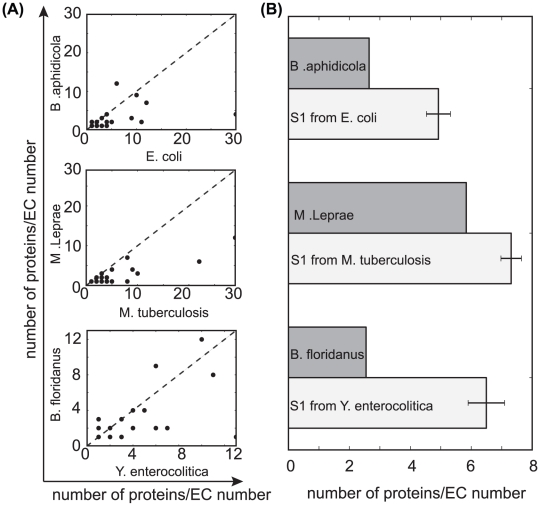
Reduction in enzymatic redundancy. (**A**) Number of proteins per E.C number in closely related organisms, defined at the most functionally specific level (fourth E.C. digit). Species are the same as in Figure 5. Note that Reduced genomes systematically code for less proteins of each E.C number, indicating loss of genetic redundancy in enzyme functions (**B**) the number of proteins per E.C. number is smaller in Reduced genomes than could be expected under a neutral scenario of gene loss (simulated according to S1), where the starting genome is that of the closest Free living organism indicated.

Finally, we used predicted enzymatic functions to further investigate the loss of functional redundancy. We considered enzyme function predicted in KEGG [57], and described by E.C. numbers. This is a hierarchical classification of enzyme function, that describes enzyme function and substrate specificity. Two proteins that have the same E.C. number have the same function. In **Figure 6A** we show that when comparing phylogenetically close Reduced and FL genomes, the former have less enzymes that map to the same predicted E.C. number, which is consistent with the notion that in reductive genome evolution there is a drive to retain a single copy of each function. This is not simply a consequence of genome reduction, as when we simulate gene loss under a neutral scenario (S1 in Figure 2), using a closely related Free Living genome as a starting point of the simulation, we always obtain artificially reduced genomes with more proteins per E.C. number, i.e. more redundant, than observed in the Reduced genomes (**Figure 6B**).

## Discussion

Our results show that organisms that suffered extensive genome reduction in response to adaptations to predictable environments maintain a higher than expected protein family and protein domain diversity, and concomitantly lost genetic redundancy.

The excess diversity at the protein family and protein domain level that characterizes the reduced genomes cannot be accounted by a neutral scenario nor does it appear to be the by-product of selection on other characters. These families observed in Reduced genomes differ from organism to organism, and only 8% of these are present in more than 90% of the organisms, suggesting that the protein family repertoires of the Reduced genomes are the product of historical contingency as well as the specific adaptive value they represent in the ecological niche occupied by each organism. Historical contingency was also observed to play an important part in theoretical studies of reductive genome evolution of metabolic pathways [44]. Interestingly, less than half of the protein families defined by essential genes in *E. coli* are kept in Reduced genomes, which clearly illustrates how different environments demand different sets of solutions, in this case protein families.

Our results suggest that while protein family diversity is preserved in genome reduction, genetic redundancy is lost. Bacterial genomes are widely reported to have smaller protein families than eukaryotes [29]–[31], relying less on genetic redundancy as a means of robustness. In fact, recently Freilich and co-workers showed that enzymes in prokaryotes are less functionally redundant than in eukaryotes [58]. Our results however suggest that free living bacteria still rely on genetic redundancy as a source of robustness. Reduced genomes have twice the number of singletons as FL, *i.e.* twice the number of genes that do not have copy backup. This is a lower bound for an estimate of the decrease in genetic redundancy in reductive genome evolution. We are not considering, for example, partial or domain redundancy [10], additional redundancy that may also be sacrificed in reductive genome evolution. One such example is that most members of the order Enterobacteriales, which includes *E. coli*, have the chaperone DnaJ as well as two proteins that share specific domains with it, CbpA and DjlA. These have been shown to be functionally redundant with DnaJ [59]. The intracellular endosymbionts *Buchnera aphidicola APS* and Candidatus *Blochmannia floridanus*, members of the same order, still have DnaJ but lost CbpA and DjlA.

It is important to note however that genetic redundancy is but one source of robustness. There is anecdotal evidence suggesting that reductive genome evolution may sacrifice other types of robustness that do not involve copy redundancy, complete or incomplete. For example, loss of network redundancy, *i.e.* alternative pathways in the synthesis of acetylCoA (two pathways in *E. coli*), was reported in the reductive evolution of *B. aphidicola* (one pathway) [44]. In another example, Cyanobacteria have an oscillator coded by three unrelated genes (KaiA, KaiB, KaiC), capable of maintaining cell cycle rhythms independently of external light-dark cycles. Members of the marine genus *Prochlorococcus*, although free living, have undergone extensive genome reduction [60], and have lost KaiA. As a consequence, the oscillator became less robust to external light cycles [61]. One promising avenue of research is then to understand to what extent other sources of robustness are affected in the reductive genome evolution.

An abundant body of theoretical work predicts that variable, unpredictable environments select for, or promote the emergence of robustness (reviewed in [11]). Abundant anecdotal examples support this prediction. For example, Sanchez-Perez and co-workers [62] proposed recently that after duplication, paralogues may retain the initial function but specialize to work under different environmental conditions. These ‘ecoparalogues’ which could still effectively compensate for each other, *i.e.* are functionally redundant, would support a link between environmental unpredictability and the emergence of robustness. They were able to find examples of proteins that are predicted to perform the same function but have different isoelectric points, and hence are predicted to operate at different ranges of salinity. Thus protein specialization under varying environments may provide the drive for the emergence of genetic redundancy. We now invert this reasoning and show that the transition to a predictable environment removes that drive, resulting in the selective loss of genetic redundancy and hence, robustness. To the best of our knowledge our results provide the first systematic description of the loss of robustness by genetic redundancy in the evolution of cellular organisms.

Redundancy is common in higher organisms that experience low mutation rates and small population sizes, and low in organisms that have high mutation rates and large population sizes [63]. Since commitment to an intracellular lifestyle is typically associated to a radical reduction in the effective population size [18]–[20] and high mutation rates [21], it would be reasonable to expect that there would be a concomitant increase in redundancy [63], [64]. This is however the opposite of what we observe – obligate parasites and endosymbionts that suffered a decrease in population size and increase in mutation rate experiencing a decrease in (genetic) redundancy. We thus provide empirical support to the notion that the predictability of the environment is of paramount importance in the evolution of redundancy. Supporting our conclusion is the observation that modularity, a characteristic of biological systems that has been linked to robustness [2], has also been shown to vary with environmental predictability, with more modular networks being found in more unpredictable environments [65]. Note that genome reduction may not be a pre-requisite for loss of genetic redundancy, as even organisms like the marine bacteria of the genus *Pirellula*, inhabiting a predictable environment, have a remarkably small number of paralogues, while retaining very large genomes [31]

Finally, many of the Reduced organisms that we studied here are causative agents of human diseases such as Lyme disease, leprosy, typhus, tularemia, pneumonia, among others. The realization that they all share a lack of robustness due to the loss of redundancy suggests new avenues for the identification of drugable targets. Instead of aiming to identify genes or pathways that are specific to the pathogenic organism, we can aim to target fragile parasite pathways in the context of robust host functions.

## Methods

### Data

The complete list of species used in this study is given as supplementary material (**Tables S3 and S4 in text S1**). It consists of 308 free living bacteria and 69 reduced genomes. Reduced genomes include obligate intracellular parasites (34 organisms) and endosymbionts (15 organisms), obtained from [15], [16], [66]. It further included parasitic bacteria like *Mycoplasma sp.*, which while not being intracellular are obligate parasites displaying signs of extreme genome reduction [51] (20 organisms). Essential genes in *E. coli* were obtained from [67] and from the PEC database (www.shigen.nig.ac.jp/ecoli/pec/). Functional class assignments were obtained from the COGs database [68], [69]. Analysis involving COGS included only genomes with more than 50% COG coverage: 176 free living and 54 reduced genomes. Functional classification with E.C numbers was obtained from KEGG [57].

### Identification of protein families

We used domain architecture as defined in the Superfamily database [32] to identify protein families. Two proteins are considered part of the same family if they display the same N- to C-terminal domain architecture, ignoring gaps as described in [70]. Domain assignments were based on Superfamily release 1.69 [71]. Sequence similarity was computed using BLAST [72] at a cutoff of E≤0.01 and orthologues were identified as reciprocal best hits [73].

### Elasticity and High pass filter

Considering a power law function *y* = γ*x* ^α^, the elasticity of *y* in relation to *x* is a constant: *(dy/dx)(x/y)* = α. The elasticity can be estimated using the linearization ln(*y*) = β1+β2ln(*x*), where β1 = ln(γ) and β2 = α. The filtered average family size is computed as (F/N)•(n/N)2, where n is the number of organisms where it appears and N the total number of organisms.

### Models of gene loss

We simulated gene loss scenarios the following way. We randomly picked one free-living genome from the set of 309 as the start point. Then we used a log-normal distribution approximated to the Reduced genome size distributions to randomly generate an end point of the simulation, *i.e.* the final size of the artificially reduced genome. We then randomly picked genes from the start genome to be “lost”, until we reached the final size. The probability of gene loss was adjusted in six alternative ways. In S1 it was totally random and represents also the background of all other scenarios. In S2 the probability of loss is made to depend linearly on the number of protein domains, *i.e.* a protein with two domains was twice as likely to be lost as a protein with a single domain. In S3 we consider essentiality a property of the family [42]. We made the probability of loss depend on the protein family distribution of known essential genes in *E. coli*. Protein families rich in essential genes (>50%) had a 2 fold decrease in the probability of loss, and protein families with less than 50% had just the random background probability of loss. In S4 we adjusted the probability of loss to the functional class distributions in the reduced genomes. Functional classes that are more frequent in Reduced genomes (**Figure S5 in Text S1**) had its probability of loss reduced to half (functional classes F,J,L,O and U), and those functional classes that are less frequent in reduced genomes had double the probability of loss (E,K,P,Q,R,S and T). In scenario S5 we used KEGG pathway assignment to predict pathway participation and considered that once a gene was lost, members of the same pathway were three times more likely to be lost afterwards. Finally, bacterial genomes are frequently organized in operons, which results in functionally related proteins being coded by genes in close proximity on the chromosome. We considered this in scenario S6 where once a gene is lost, the probability of its adjacent genes being lost afterwards increases twofold.

### Estimates of probability of gene loss

We estimate the probability of losing proteins in a given family Ploss as the ratio between the total number of elements lost in the family over the size of that family in FL. P_loss_(F_FL_) = (F_FL_−F_Reduced_)/F_FL_. F_FL_ and F_Reduced_ are the total number of elements of the family in each class of genomes; for this analysis we only considered families that appear in 90% or more organisms in both classes.

## Supporting Information

Text S1 Supplementary data and methods.
(1.13 MB PDF)
