# Supplementary material for: Loss of Genetic Redundancy in Reductive Genome Evolution
Source: PLoS Comput Biol. 2011 Feb 17;7(2):e1001082. doi: 10.1371/journal.pcbi.1001082 (PMC3040653; doi:10.1371/journal.pcbi.1001082)
Supplement: Text S1 — Supplementary data and methods. (1.13 MB PDF) [file pcbi.1001082.s001.pdf]

Supplementary material for

Loss of genetic redundancy in reductive genome evolution

André Mendonça, Renato J. Alves and José B. Pereira-Leal

1. Supplementary information

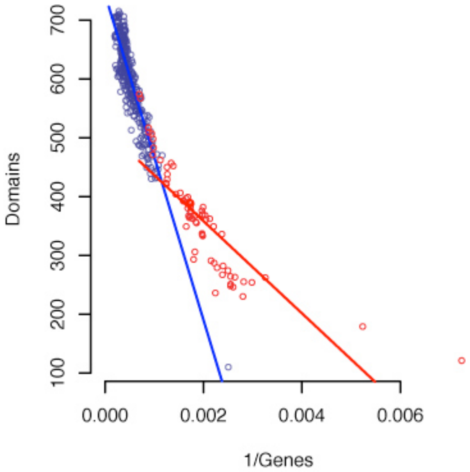

Figure S1 - Reduced genomes have a higher number of domains per gene than FL. Plotted are the domains vs. number of genes for FL (Blue) and Reduced genomes (red). Solid lines represent a fit by an inverse function. The slopes and intersects are statistically different ($p<1.x10^{-66}$, $p<3.72x10^{-65}$, respectively, *t* test; R^2=0.915).

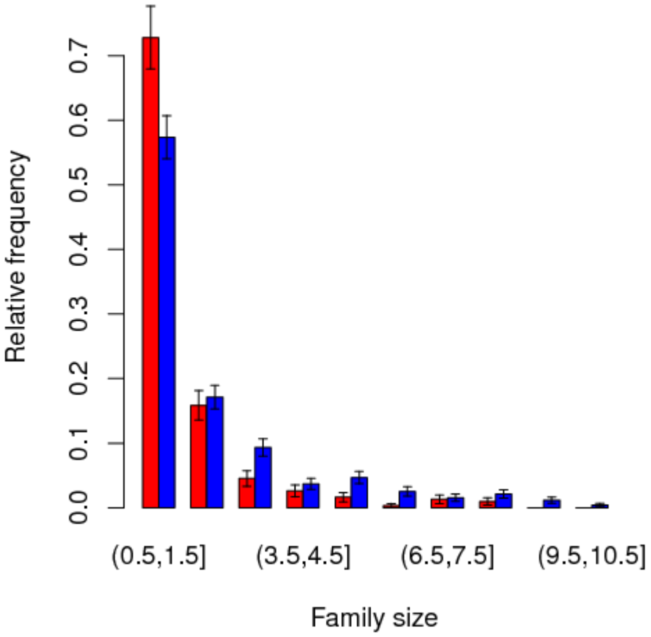

Figure S2 - Reduced Actinomycetales genomes have a higher number of singletons and smaller number of protein families with more than one element (N_{FL}=27; N_R=2; p= 1.064e-07). This corresponds to the analysis shown on figure 1C, performed on a subset of organisms (see tables M1 and M2)

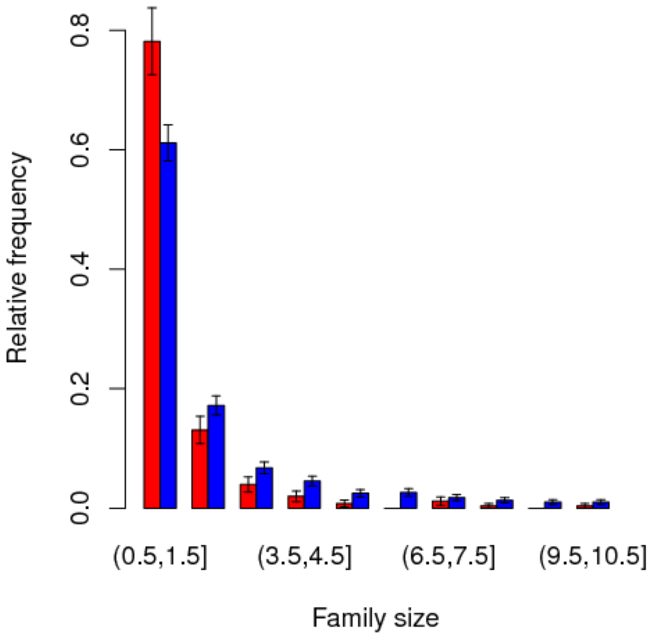

Figure S3 - Reduced Enterobacteriaceae genomes have a higher number of singletons and smaller number of protein families with more than one element (N_{FL}=10; N_R=8; p= 3.815e^{-06}). This corresponds to the analysis shown on figure 1C, performed on a subset of organisms (see tables M1 and M2)

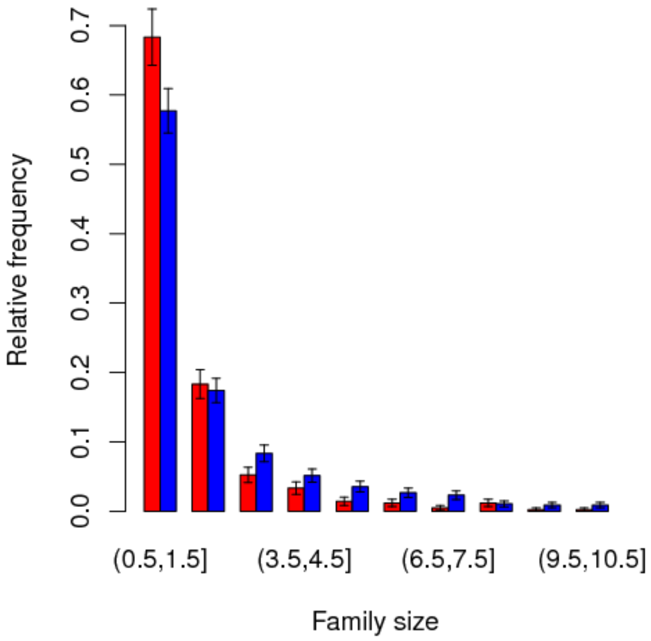

Figure S4 - Reduced Rhizobiales genomes have a higher number of singletons and smaller number of protein families with more than one element (N_{FL}=19; N_R=3; p= 1.695e^{-07}). This corresponds to the analysis shown on figure 1C, performed on a subset of organisms (see tables M1 and M2)

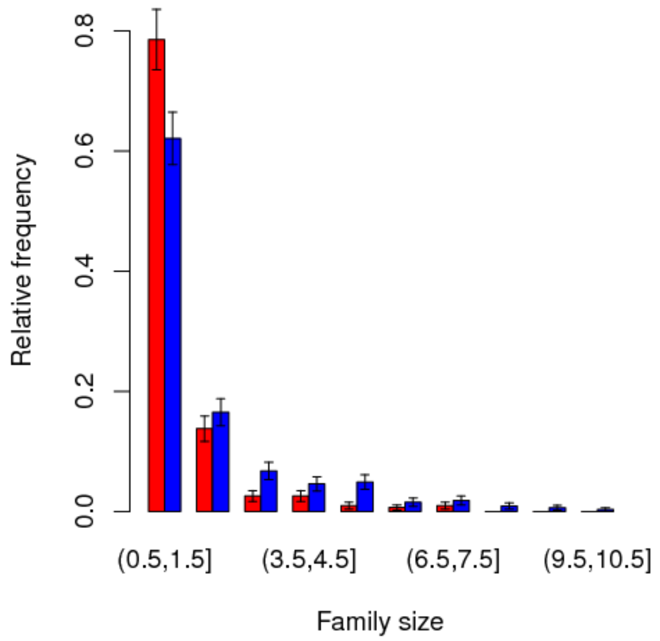

Figure S5 - Reduced Spirochaetales genomes have a higher number of singletons and smaller number of protein families with more than one element (N_{FL}=4; N_R=3; p= 1.266e^{-05}). This corresponds to the analysis shown on figure 1C, performed on a subset of organisms (see tables M1 and M2)

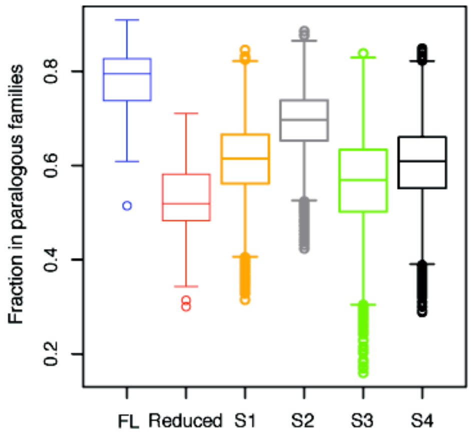

Figure S6 – Free-living organisms have significantly more genes in paralogous families, *i.e.* family size≥2, than reduced genomes (FL= 0.784 ± 0.063; Reduced= 0.546 ± 0.066, p<2.2x10^{-16}; Mann-Whitney). Reduced genomes also have less genes in paralogous families than expected under any of the simulated scenarios. (p_{S1}=1.5x10^{-5}, p_{S2}=2.2x10^{-6}, p_{S3}=0.0002, p_{S4}=3.0x10^{-13}, Wilcoxon rank sum test)

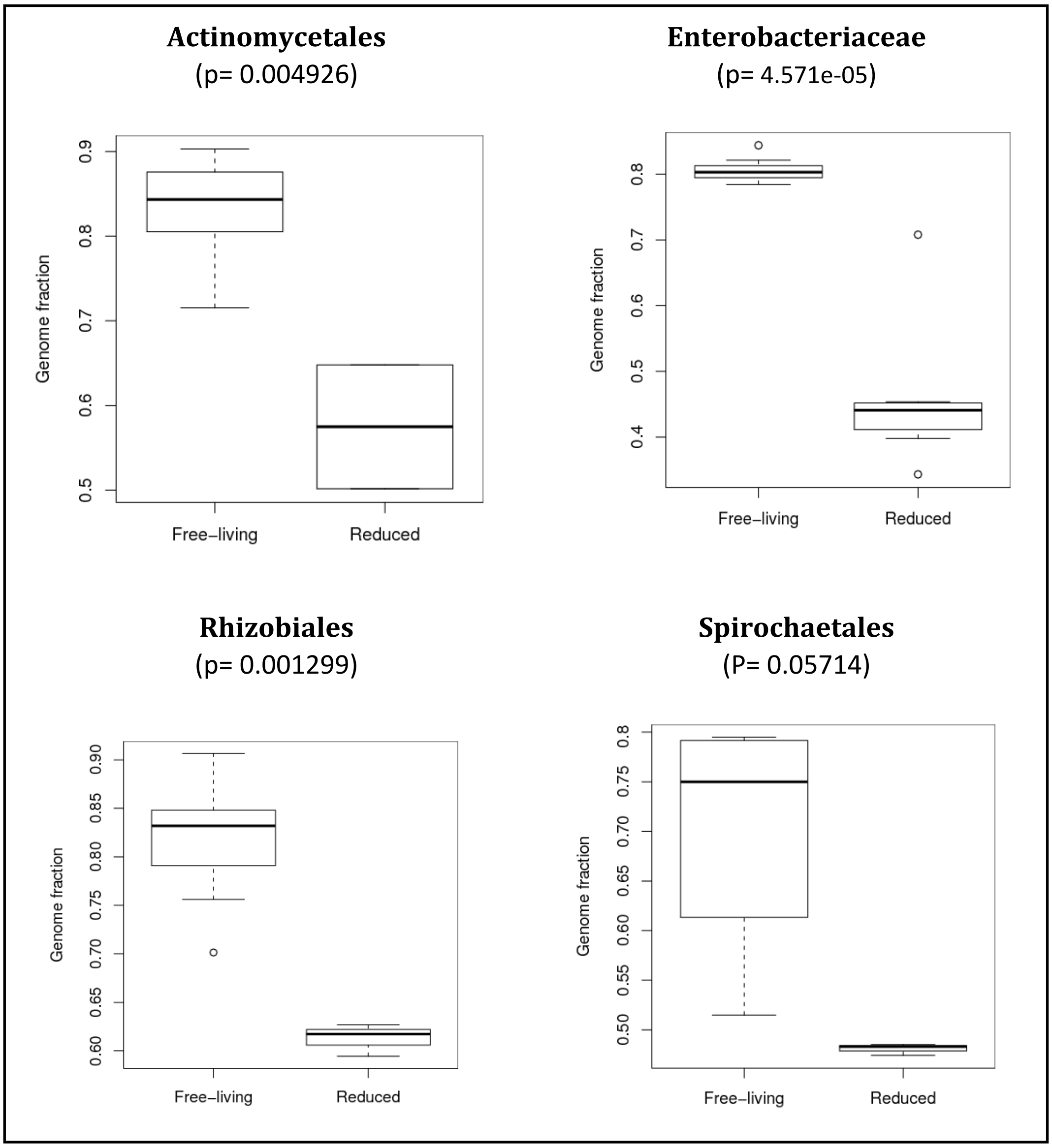

Figure S7 - Relative frequency of genes in paralogous families in FL, Reduced, by taxonomic group. P value calculated with the Wilcoxon rank sum test with continuity correction. This corresponds to the analysis shown in figure S6 for a subset of phylogenetically close organisms (see tables M1 and M2)

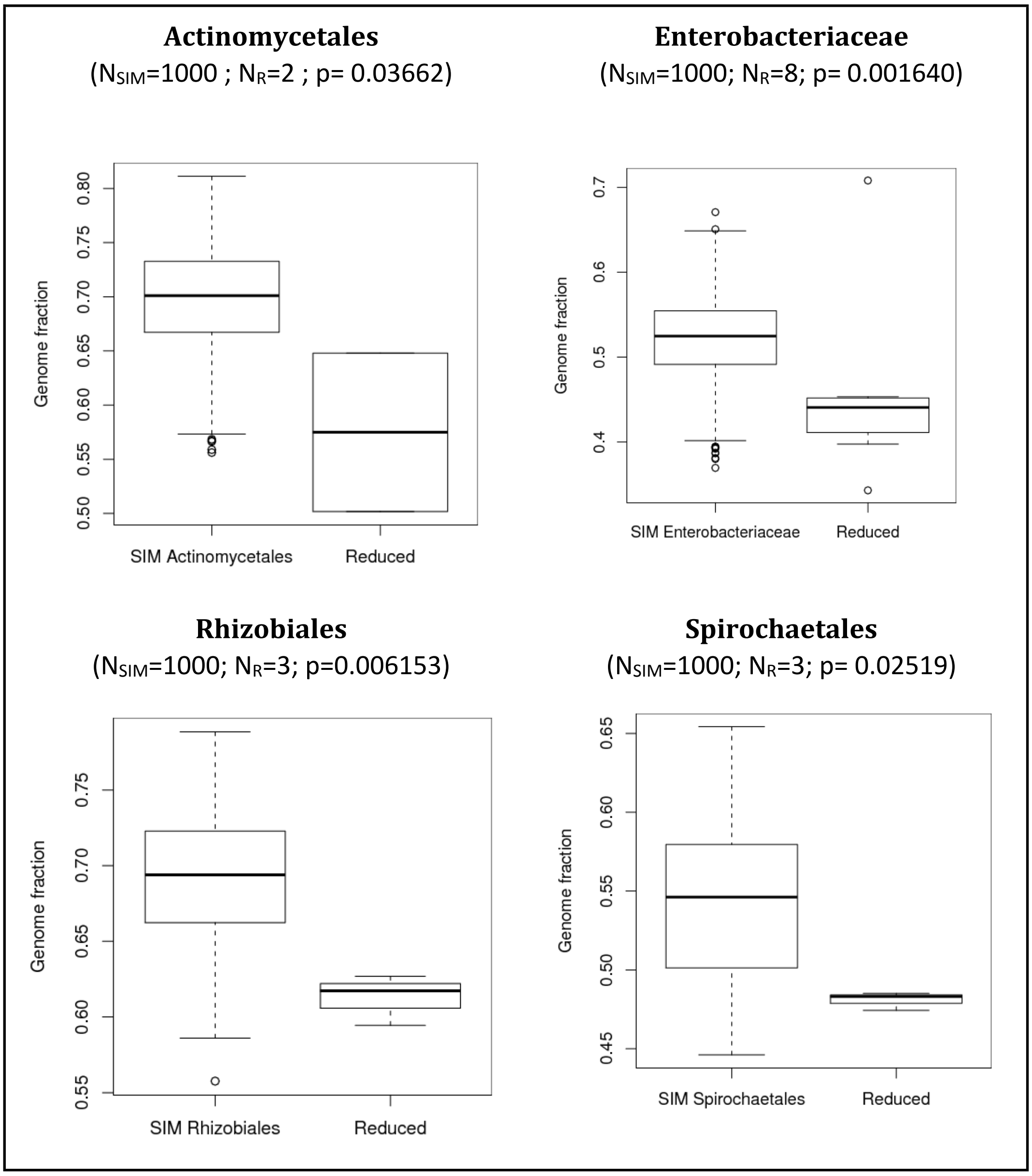

Figure S8 - Fraction of singletons in the genomes of simulated and Reduced genomes. P value calculated with the Wilcoxon rank sum test with continuity correction. N_{SIM}= numer of simulated genomes; N_R=number of reduced genomes. This corresponds to the analysis shown in figure 2C for a subset of phylogenetically close organisms (see tables M1 and M2)

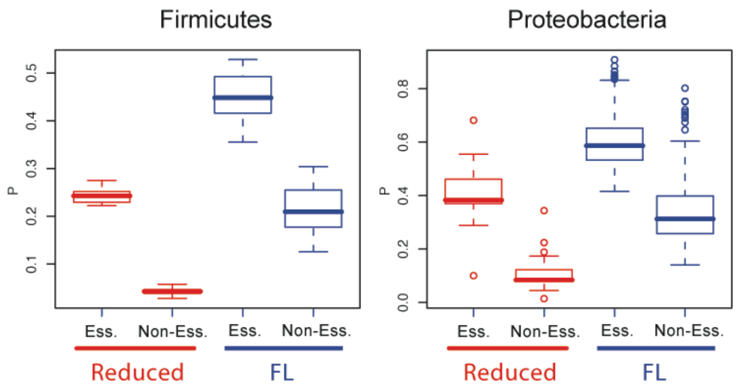

Figure S9 - Probability of conservation of genes according to their deletion phenotype in *E. coli* (Essential and Non-Essential) (Krylov et al., 2003), in free living (Blue) and reduced genome organisms (Red). Orthology was determined by BLAST bi-directional best hit at a cut off of 10^{-2} and is only show for the Phyla for which there were sufficient reduced and free living to allow statistical analysis. As expected, essential genes are more likely to be conserved in free-living organisms than non-essential genes . We observed that this is also the case in organisms that suffered extensive genome reduction, at, $p_{Firmicutes}=6.9\times10^{-7}$, $p_{Proteobacteria}=9.6\times10^{-14}$ (Mann-Whitney)

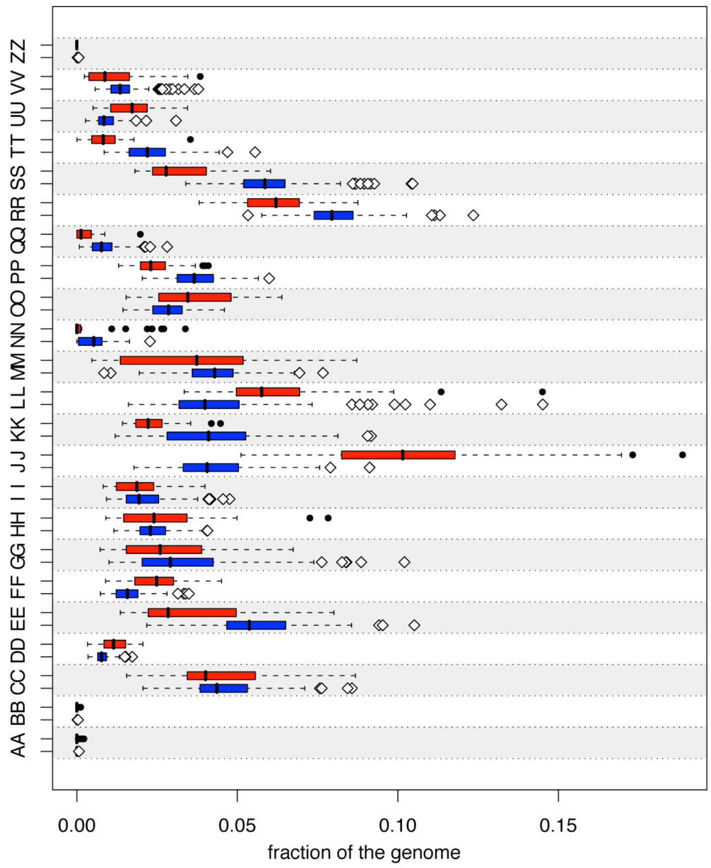

Figure S10 - COG functional class distribution in Free living (blue) and reduced (red) genomes.

Table S1 – Statistics of the COG functional class distributions in FL and Reduced. f is the average frequency of the functional class, S the standard deviation, FL and R stand for free living and reduced, respectively. Functional classes whose frequency is significantly different in FL and R are highlighted in the rejection column.

	$\bar{f}^{FL}$	s^{FL}	$\bar{f}^{R}$	s^{R}	t	p- value	Rejection	Sign
A	0.01%	0.02%	0.03%	0.07%	-2.28	0.026		
B	0.00%	0.00%	0.01%	0.04%	-2.54	0.014		
C	4.58%	1.18%	4.46%	1.70%	0.48	0.634		
D	0.81%	0.24%	1.16%	0.41%	-6.11	6.42E-08	*	+
E	5.56%	1.33%	3.67%	1.85%	6.98	1.30E-09	*	-
F	1.63%	0.53%	2.46%	0.86%	-6.81	3.65E-09	*	+
G	3.33%	1.73%	2.94%	1.66%	1.50	0.137		
H	2.44%	0.62%	2.69%	1.52%	-1.20	0.235		
I	2.12%	0.79%	1.98%	0.88%	1.01	0.315		
J	4.27%	1.32%	10.43%	3.15%	-14.02	2.06E-20	*	+
K	4.19%	1.66%	2.35%	0.68%	11.83	4.41E-25	*	-
L	4.48%	2.34%	6.30%	1.97%	-5.70	1.16E-07	*	+
M	4.28%	0.99%	3.61%	2.46%	1.96	0.055		
N	0.51%	0.42%	0.37%	0.85%	1.20	0.235		
O	2.81%	0.66%	3.67%	1.32%	-4.63	1.93E-05	*	+
P	3.69%	0.78%	2.42%	0.65%	11.88	4.49E-21	*	-
Q	0.86%	0.50%	0.24%	0.34%	10.38	1.18E-18	*	-
R	8.02%	1.05%	6.09%	1.13%	11.13	3.89E-18	*	-
S	5.96%	1.27%	3.21%	1.11%	15.30	8.05E-28	*	-
T	2.27%	0.83%	0.85%	0.58%	14.07	1.50E-27	*	-
U	0.92%	0.38%	1.71%	0.77%	-7.29	7.23E-10	*	+
V	1.44%	0.56%	1.15%	0.94%	2.17	0.034		
Z	0.00%	0.01%	0.00%	0.00%	1.62	0.106		
unk	35.83%	7.00%	38.18%	9.84%	-1.63	0.107		

Table S2 – COG functional classes

	Function
A	RNA processing and modification
B	Chromatin structure and dynamics
C	Energy production and conversion
D	Cell cycle control, mitosis and meiosis
E	Amino acid transport and metabolism
F	Nucleotide transport and metabolism
G	Carbohydrate transport and metabolism
H	Coenzyme transport and metabolism
I	Lipid transport and metabolism
J	Translation
K	Transcription
L	Replication, recombination and repair
M	Cell wall/membrane biogenesis
N	Cell motility
O	Posttranslational modification, protein turnover, chaperones
P	Inorganic ion transport and metabolism
Q	Secondary metabolites biosynthesis, transport and catabolism
R	General function prediction only
S	Function unknown
T	Signal transduction mechanisms
U	Intracellular trafficking and secretion
V	Defense mechanisms
W	Extracellular structures
Z	Cytoskeleton

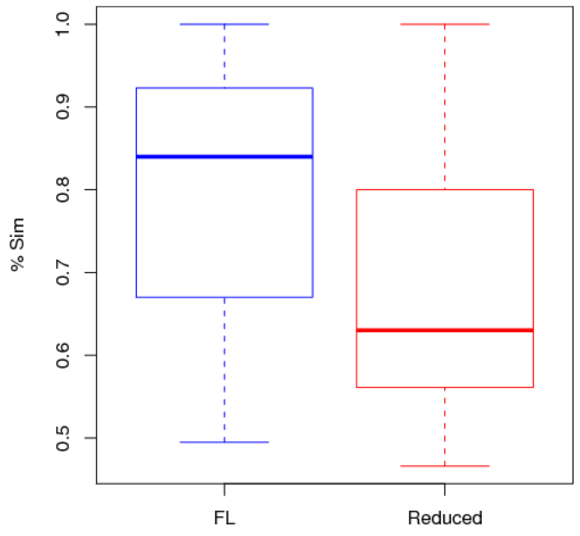

Figure S11 - The similarity between the closest paralogues in the similarity network in each protein family in families that have not reduced in size is not statistically different (p.value: 0.14, Wilcoxon)

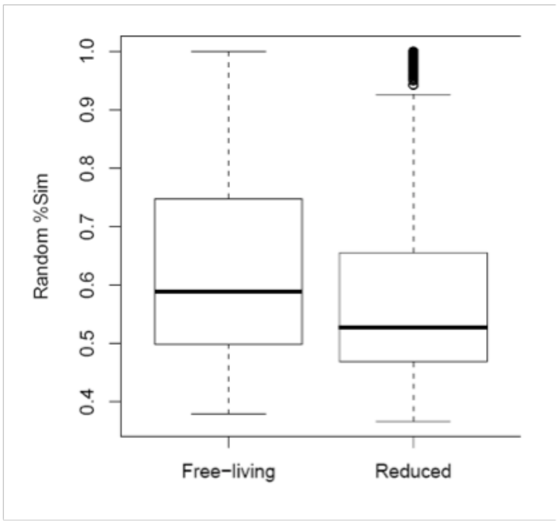

Figure S12 - Family size does not affect similarity difference. This analysis aims to address a the possibility that the difference observed in figure 5 may be due to Reduced organisms having smaller families. This is a well known problem of extreme statistics: the minimum distance between two members in a network is expected, by chance alone, to be smaller if the number of members is larger. To control for this we conducted a new experiment, which corresponds to artificially setting all family sizes to two elements only, thus controlling for family size. We randomly pick a distance from a family in Reduced, and then from the same family in Free-Living (blind to the minimum value) - we do this 10,000 times, with repetition, randomly sampling protein families. In this way we control for family size, as the numbers of elements are now two in each family group, and thus the same. However, one group (Reduced) suffered gene loss, whereas the other (FL), did not. If the observation reported in figure 5 is artifactual, i.e that we are not actively losing close paralogues, then we expect to see no difference in this scenario. However, what we observe is that Reduced families of 'size two' are significantly less similar (p=0.018, figure S13) than free living families of the same size. This argues that what we observe is not a statistical effect obtained by chance but in fact a loss of the closest family members in a network by Reduced organisms.

Table S3 – Reduced genomes used in this study. Lifestyle codes are P – parasite, pathogen, E – endosymbionte, X - extracellular

Species	Phylum	Lifestyle
Anaplasma marginale	Proteobacteria	P
Anaplasma phagocytophilum	Proteobacteria	P
Aster yellows witches-broom phytoplasma	Firmicutes	P , X(?)
Bartonella bacilliformis	Proteobacteria	P
Bartonella henselae	Proteobacteria	P
Bartonella quintana	Proteobacteria	P
Baumannia cicadellinicola	Proteobacteria	E
Borrelia afzelii	Spirochaetes	P
Borrelia burgdorferi	Spirochaetes	P, X
Borrelia garinii	Spirochaetes	P, X
Buchnera aphidicola APS	Proteobacteria	E
Buchnera aphidicola Bp	Proteobacteria	E
Buchnera aphidicola Cc	Proteobacteria	E
Buchnera aphidicola Sg	Proteobacteria	E
Candidatus Blochmannia floridanus	Proteobacteria	E
Candidatus Blochmannia pennsylvanicus	Proteobacteria	E

Candidatus Carsonella ruddii	Proteobacteria	E
Candidatus Protochlamydia amoebophila	Chlamydiae	P
Candidatus Ruthia magnifica	Proteobacteria	E
Candidatus Sulcia muelleri	Bacteroidetes	E
Candidatus Vesicomyosocius okutanii	Proteobacteria	E, X(?)
Chlamydia muridarum	Chlamydiae	P
Chlamydia trachomatis	Chlamydiae	P
Chlamydophila abortus	Chlamydiae	P
Chlamydophila caviae	Chlamydiae	P
Chlamydophila felis	Chlamydiae	P
Chlamydophila pneumoniae	Chlamydiae	P
Coxiella burnetii	Proteobacteria	P
Ehrlichia canis	Proteobacteria	P, X(?)
Ehrlichia chaffeensis	Proteobacteria	P
Ehrlichia ruminantium	Proteobacteria	P
Francisella tularensis</i> ssp. <i>holarctica	Proteobacteria	P
Francisella tularensis</i> ssp. <i>novicida	Proteobacteria	P
Francisella tularensis</i> ssp. <i>tularensis	Proteobacteria	P
Lawsonia intracellularis	Proteobacteria	P
Mesoplasma florum	Firmicutes	P
Mycobacterium leprae	Actinobacteria	P, X
Mycoplasma agalactiae	Firmicutes	P, X
Mycoplasma capricolum</i> ssp. <i>capricolum	Firmicutes	P, X
Mycoplasma gallisepticum	Firmicutes	P, X
Mycoplasma genitalium	Firmicutes	P, X
Mycoplasma hyopneumoniae 232	Firmicutes	P, X
Mycoplasma hyopneumoniae 7448	Firmicutes	P, X
Mycoplasma hyopneumoniae J	Firmicutes	P, X
Mycoplasma mobile	Firmicutes	P, X
Mycoplasma mycoides</i> ssp. <i>mycoides	Firmicutes	P, X
Mycoplasma penetrans	Firmicutes	P, X
Mycoplasma pneumoniae	Firmicutes	P, X
Mycoplasma pulmonis	Firmicutes	P, X
Mycoplasma synoviae	Firmicutes	P, X
Neorickettsia sennetsu	Proteobacteria	P
Onion yellows phytoplasma	Firmicutes	P
Orientia tsutsugamushi	Proteobacteria	P
Polynucleobacter	Proteobacteria	P
Rickettsia akari	Proteobacteria	P
Rickettsia bellii	Proteobacteria	P
Rickettsia canadensis	Proteobacteria	P
Rickettsia conorii	Proteobacteria	P
Rickettsia felis	Proteobacteria	P
Rickettsia massiliae	Proteobacteria	P
Rickettsia prowazekii	Proteobacteria	P
Rickettsia rickettsii	Proteobacteria	P
Rickettsia typhi	Proteobacteria	P
Sodalis glossinidius morsitans	Proteobacteria	E
Tropheryma whipplei	Actinobacteria	P, X
Ureaplasma parvum	Firmicutes	P, X
Wigglesworthia glossinidia	Proteobacteria	E
Wolbachia</i> endosymbiont of <i>Drosophila melanogaster	Proteobacteria	E
Wolbachia</i> endosymbiont TRS of <i>Brugia malayi	Proteobacteria	E

Table S4– free living genomes used in this study

Species	**Phylum**
Acidobacteria bacterium	Acidobacteria
Acidothermus cellulolyticus	Actinobacteria
Acidovorax	Proteobacteria
Acidovorax avenae</i> ssp. <i>citrulli	Proteobacteria
Acinetobacter baumannii	Proteobacteria
Actinobacillus pleuropneumoniae	Proteobacteria
Actinobacillus succinogenes	Proteobacteria
Aeromonas hydrophila</i> ssp. <i>hydrophila	Proteobacteria
Aeromonas salmonicida</i> ssp. <i>salmonicida	Proteobacteria
Alcanivorax borkumensis	Proteobacteria
Alkalilimnicola ehrlichei	Proteobacteria
Alkaliphilus metalliredigens	Firmicutes
Alkaliphilus oremlandii	Firmicutes
Anabaena variabilis	Cyanobacteria
Anaeromyxobacter	Proteobacteria
Anaeromyxobacter dehalogenans	Proteobacteria
Aquifex aeolicus	Aquificae
Arcobacter butzleri	Proteobacteria
Arthrobacter	Actinobacteria
Arthrobacter aurescens	Actinobacteria
Azoarcus	Proteobacteria
Azorhizobium caulinodans	Proteobacteria
Bacillus amyloliquefaciens	Firmicutes
Bacillus cereus	Firmicutes
Bacillus cereus</i> ssp. <i>cytotoxis	Firmicutes
Bacillus clausii	Firmicutes
Bacillus halodurans	Firmicutes
Bacillus licheniformis	Firmicutes
Bacillus pumilus	Firmicutes
Bacillus subtilis</i> ssp. <i>subtilis	Firmicutes
Bacillus thuringiensis	Firmicutes
Bacillus thuringiensis</i> ser. <i>konkukian	Firmicutes
Bacillus weihenstephanensis	Firmicutes
Bacteroides fragilis	Bacteroidetes
Bacteroides vulgatus	Bacteroidetes
Bartonella tribocorum	Proteobacteria
Bdellovibrio bacteriovorus	Proteobacteria
Bifidobacterium adolescentis	Actinobacteria
Bordetella petrii	Proteobacteria
Bradyrhizobium	Proteobacteria
Bradyrhizobium japonicum	Proteobacteria
Brucella canis	Proteobacteria
Brucella melitensis	Proteobacteria
Brucella ovis	Proteobacteria
Brucella suis	Proteobacteria
Burkholderia	Proteobacteria
Burkholderia cenocepacia	Proteobacteria
Burkholderia cepacia	Proteobacteria
Burkholderia multivorans	Proteobacteria
Burkholderia thailandensis	Proteobacteria
Burkholderia vietnamiensis	Proteobacteria
Burkholderia xenovorans	Proteobacteria
Caldicellulosiruptor saccharolyticus	Firmicutes
Campylobacter concisus	Proteobacteria

Campylobacter curvus	Proteobacteria
Campylobacter fetus</i> ssp. <i>fetus	Proteobacteria
Campylobacter hominis	Proteobacteria
Campylobacter jejuni	Proteobacteria
Campylobacter jejuni</i> ssp. <i>doylei	Proteobacteria
Campylobacter jejuni</i> ssp. <i>jejuni	Proteobacteria
Candidatus Pelagibacter ubique	Proteobacteria
Carboxydothermus hydrogenoformans	Firmicutes
Caulobacter crescentus	Proteobacteria
Chlorobium chlorochromatii	Chlorobi
Chlorobium phaeobacteroides	Chlorobi
Chlorobium tepidum	Chlorobi
Chloroflexus aurantiacus	Chloroflexi
Chromobacterium violaceum	Proteobacteria
Chromohalobacter salexigens	Proteobacteria
Citrobacter koseri	Proteobacteria
Clavibacter michiganensis</i> ssp. <i>michiganensis	Actinobacteria
Clostridium acetobutylicum	Firmicutes
Clostridium beijerinckii	Firmicutes
Clostridium botulinum	Firmicutes
Clostridium difficile	Firmicutes
Clostridium kluyveri	Firmicutes
Clostridium novyi	Firmicutes
Clostridium perfringens	Firmicutes
Clostridium phytofermentans	Firmicutes
Clostridium tetani	Firmicutes
Clostridium thermocellum	Firmicutes
Colwellia psychrerythraea	Proteobacteria
Corynebacterium efficiens	Actinobacteria
Corynebacterium glutamicum	Actinobacteria
Corynebacterium jeikeium	Actinobacteria
Cytophaga hutchinsonii	Bacteroidetes
Dechloromonas aromatica	Proteobacteria
Dehalococcoides	Chloroflexi
Dehalococcoides ethenogenes	Chloroflexi
Deinococcus geothermalis	Deinococcus-Thermus
Deinococcus radiodurans	Deinococcus-Thermus
Delftia acidovorans	Proteobacteria
Desulfitobacterium hafniense	Firmicutes
Desulfococcus oleovorans	Proteobacteria
Desulfotalea psychrophila	Proteobacteria
Desulfotomaculum reducens	Firmicutes
Desulfovibrio desulfuricans	Proteobacteria
Desulfovibrio vulgaris</i> ssp. <i>vulgaris	Proteobacteria
Dichelobacter nodosus	Proteobacteria
Dinoroseobacter shibae	Proteobacteria
Enterobacter	Proteobacteria
Enterobacter sakazakii	Proteobacteria
Erwinia carotovora</i> ssp. <i>atroseptica	Proteobacteria
Erythrobacter litoralis	Proteobacteria
Fervidobacterium nodosum	Thermotogae
Flavobacterium johnsoniae	Bacteroidetes
Flavobacterium psychrophilum	Bacteroidetes
Frankia	Actinobacteria
Frankia alni	Actinobacteria
Fusobacterium nucleatum</i> ssp. <i>nucleatum	Fusobacteria
Geobacillus kaustophilus	Firmicutes
Geobacillus thermodenitrificans	Firmicutes

Geobacter metallireducens	Proteobacteria
Geobacter sulfurreducens	Proteobacteria
Geobacter uraniumreducens	Proteobacteria
Gloeobacter violaceus	Cyanobacteria
Gluconacetobacter diazotrophicus	Proteobacteria
Gluconobacter oxydans	Proteobacteria
Gramella forsetii	Bacteroidetes
Granulibacter bethesdensis	Proteobacteria
Haemophilus influenzae	Proteobacteria
Haemophilus somnus	Proteobacteria
Hahella chejuensis	Proteobacteria
Halorhodospira halophila	Proteobacteria
Herminiimonas arsenicoxydans	Proteobacteria
Herpetosiphon aurantiacus	Chloroflexi
Hyphomonas neptunium	Proteobacteria
Idiomarina loihiensis	Proteobacteria
Jannaschia	Proteobacteria
Janthinobacterium	Proteobacteria
Kineococcus radiotolerans	Actinobacteria
Klebsiella pneumoniae</i> ssp. <i>pneumoniae	Proteobacteria
Lactobacillus acidophilus	Firmicutes
Lactobacillus brevis	Firmicutes
Lactobacillus casei	Firmicutes
Lactobacillus delbrueckii</i> ssp. <i>bulgaricus	Firmicutes
Lactobacillus gasseri	Firmicutes
Lactobacillus helveticus	Firmicutes
Lactobacillus plantarum	Firmicutes
Lactobacillus reuteri	Firmicutes
Lactobacillus sakei</i> ssp. <i>sakei	Firmicutes
Lactobacillus salivarius</i> ssp. <i>salivarius	Firmicutes
Lactococcus lactis</i> ssp. <i>cremoris	Firmicutes
Lactococcus lactis</i> ssp. <i>lactis	Firmicutes
Legionella pneumophila	Proteobacteria
Legionella pneumophila</i> ssp. <i>pneumophila	Proteobacteria
Leifsonia xyli</i> ssp. <i>xyli	Actinobacteria
Leptospira borgpetersenii	Spirochaetes
Leptospira interrogans	Spirochaetes
Leuconostoc mesenteroides</i> ssp. <i>mesenteroides	Firmicutes
Listeria innocua	Firmicutes
Listeria monocytogenes	Firmicutes
Listeria welshimeri	Firmicutes
Magnetospirillum magneticum	Proteobacteria
Maricaulis maris	Proteobacteria
Marinobacter aquaeolei	Proteobacteria
Marinomonas	Proteobacteria
Mesorhizobium	Proteobacteria
Mesorhizobium loti	Proteobacteria
Methylibium petroleiphilum	Proteobacteria
Methylobacillus flagellatus	Proteobacteria
Methylobacterium extorquens	Proteobacteria
Methylococcus capsulatus	Proteobacteria
Moorella thermoacetica	Firmicutes
Mycobacterium	Actinobacteria
Mycobacterium avium	Actinobacteria
Mycobacterium gilvum	Actinobacteria
Mycobacterium smegmatis	Actinobacteria
Mycobacterium ulcerans	Actinobacteria
Mycobacterium vanbaalenii	Actinobacteria

Myxococcus xanthus	Proteobacteria
Neisseria gonorrhoeae	Proteobacteria
Nitratiruptor	Proteobacteria
Nitrobacter hamburgensis	Proteobacteria
Nitrobacter winogradskyi	Proteobacteria
Nitrosococcus oceani	Proteobacteria
Nitrosomonas europaea	Proteobacteria
Nitrosomonas eutropha	Proteobacteria
Nitrosospira multiformis	Proteobacteria
Nocardia farcinica	Actinobacteria
Nocardioides	Actinobacteria
Nostoc	Cyanobacteria
Novosphingobium aromaticivorans	Proteobacteria
Oceanobacillus iheyensis	Firmicutes
Ochrobactrum anthropi	Proteobacteria
Oenococcus oeni	Firmicutes
Parabacteroides distasonis	Bacteroidetes
Paracoccus denitrificans	Proteobacteria
Parvibaculum lavamentivorans	Proteobacteria
Pasteurella multocida</i> ssp. <i>multocida	Proteobacteria
Pediococcus pentosaceus	Firmicutes
Pelobacter carbinolicus	Proteobacteria
Pelobacter propionicus	Proteobacteria
Pelodictyon luteolum	Chlorobi
Pelotomaculum thermopropionicum	Firmicutes
Petrotoga mobilis	Thermotogae
Photobacterium profundum	Proteobacteria
Photorhabdus luminescens</i> ssp. <i>laumondii	Proteobacteria
Polaromonas	Proteobacteria
Polaromonas naphthalenivorans	Proteobacteria
Prochlorococcus marinus	Cyanobacteria
Prochlorococcus marinus</i> ssp. <i>marinus	Cyanobacteria
Prochlorococcus marinus</i> ssp. <i>pastoris	Cyanobacteria
Prosthecochloris vibrioformis	Chlorobi
Pseudoalteromonas atlantica	Proteobacteria
Pseudoalteromonas haloplanktis	Proteobacteria
Pseudomonas aeruginosa	Proteobacteria
Pseudomonas entomophila	Proteobacteria
Pseudomonas fluorescens	Proteobacteria
Pseudomonas mendocina	Proteobacteria
Pseudomonas putida	Proteobacteria
Pseudomonas stutzeri	Proteobacteria
Pseudomonas syringae</i> pv. <i>phaseolicola	Proteobacteria
Pseudomonas syringae</i> pv. <i>syringae	Proteobacteria
Pseudomonas syringae</i> pv. <i>tomato	Proteobacteria
Psychrobacter	Proteobacteria
Psychrobacter arcticus	Proteobacteria
Psychrobacter cryohalolentis	Proteobacteria
Psychromonas ingrahamii	Proteobacteria
Ralstonia eutropha	Proteobacteria
Ralstonia metallidurans	Proteobacteria
Renibacterium salmoninarum	Actinobacteria
Rhizobium etli	Proteobacteria
Rhizobium leguminosarum</i> bv. <i>viciae	Proteobacteria
Rhodobacter sphaeroides	Proteobacteria
Rhodococcus	Actinobacteria
Rhodoferax ferrireducens	Proteobacteria
Rhodopirellula baltica	Planctomycetes

Rhodopseudomonas palustris	Proteobacteria
Rhodospirillum rubrum	Proteobacteria
Roseiflexus	Chloroflexi
Roseiflexus castenholzii	Chloroflexi
Roseobacter denitrificans	Proteobacteria
Rubrobacter xylanophilus	Actinobacteria
Saccharophagus degradans	Proteobacteria
Saccharopolyspora erythraea	Actinobacteria
Salinibacter ruber	Bacteroidetes
Salinispora arenicola	Actinobacteria
Salinispora tropica	Actinobacteria
Salmonella enterica</i> ssp. <i>arizonae	Proteobacteria
Salmonella enterica</i> ssp. <i>enterica	Proteobacteria
Serratia proteamaculans	Proteobacteria
Shewanella	Proteobacteria
Shewanella amazonensis	Proteobacteria
Shewanella baltica	Proteobacteria
Shewanella denitrificans	Proteobacteria
Shewanella frigidimarina	Proteobacteria
Shewanella loihica	Proteobacteria
Shewanella oneidensis	Proteobacteria
Shewanella pealeana	Proteobacteria
Shewanella putrefaciens	Proteobacteria
Shewanella sediminis	Proteobacteria
Shigella boydii	Proteobacteria
Shigella dysenteriae	Proteobacteria
Shigella sonnei	Proteobacteria
Silicibacter	Proteobacteria
Silicibacter pomeroyi	Proteobacteria
Sinorhizobium medicae	Proteobacteria
Sinorhizobium meliloti	Proteobacteria
Solibacter usitatus	Acidobacteria
Sorangium cellulosum</i> So <i>ce	Proteobacteria
Sphingomonas wittichii	Proteobacteria
Sphingopyxis alaskensis	Proteobacteria
Staphylococcus aureus	Firmicutes
Staphylococcus aureus</i> ssp. <i>aureus	Firmicutes
Staphylococcus haemolyticus	Firmicutes
Staphylococcus saprophyticus</i> ssp. <i>saprophyticus	Firmicutes
Streptococcus gordonii	Firmicutes
Streptococcus sanguinis	Firmicutes
Streptococcus suis	Firmicutes
Streptococcus thermophilus	Firmicutes
Streptomyces avermitilis	Actinobacteria
Streptomyces coelicolor	Actinobacteria
Sulfurovum	Proteobacteria
Symbiobacterium thermophilum	Actinobacteria
Synechococcus	Cyanobacteria
Synechococcus elongatus	Cyanobacteria
Synechocystis	Cyanobacteria
Syntrophobacter fumaroxidans	Proteobacteria
Syntrophomonas wolfei</i> ssp. <i>wolfei	Firmicutes
Syntrophus aciditrophicus	Proteobacteria
Thermoanaerobacter tengcongensis	Firmicutes
Thermobifida fusca	Actinobacteria
Thermosipho melanesiensis	Thermotogae
Thermosynechococcus elongatus	Cyanobacteria
Thermotoga lettingae	Thermotogae

Thermotoga maritima	Thermotogae
Thermotoga petrophila	Thermotogae
Thermus thermophilus	Deinococcus-Thermus
Thiobacillus denitrificans	Proteobacteria
Thiomicrospira crunogena	Proteobacteria
Thiomicrospira denitrificans	Proteobacteria
Treponema denticola	Spirochaetes
Treponema pallidum</i> ssp. <i>pallidum	Spirochaetes
Trichodesmium erythraeum	Cyanobacteria
Verminephrobacter eiseniae	Proteobacteria
Vibrio cholerae	Proteobacteria
Vibrio cholerae</i> O1 biov. <i>eltor	Proteobacteria
Vibrio fischeri	Proteobacteria
Vibrio harveyi	Proteobacteria
Xanthobacter autotrophicus	Proteobacteria
Xanthomonas axonopodis</i> pv. <i>citri	Proteobacteria
Xanthomonas campestris</i> pv. <i>campestris	Proteobacteria
Xanthomonas campestris</i> pv. <i>vesicatoria	Proteobacteria
Xanthomonas oryzae</i> pv. <i>oryzae	Proteobacteria
Yersinia enterocolitica</i> ssp. <i>enterocolitica	Proteobacteria
Yersinia pseudotuberculosis	Proteobacteria
Zymomonas mobilis</i> ssp. <i>mobilis	Proteobacteria

2. References

Krylov, D. M., Wolf, Y. I., Rogozin, I. B., and Koonin, E. V. (2003). Gene loss, protein sequence divergence, gene dispensability, expression level, and interactivity are correlated in eukaryotic evolution. Genome research *13*, 2229-2235.
